# Single‐cell RNA sequencing reveals cell immune status and dysregulated monocytes in patients with myasthenia gravis

**DOI:** 10.1002/cti2.70052

**Published:** 2025-10-05

**Authors:** Yufan Guo, Yu Gu, Yuting Jin, Xintao Wu, Yuting Lou, Pu Miao, Ye Wang, Bijun Zhang, Xueting Lin, Chudi Zhang, Jianhua Feng

**Affiliations:** ^1^ Department of Pediatrics The Second Affiliated Hospital of Zhejiang University School of Medicine Hangzhou China; ^2^ Zhejiang University School of Medicine Hangzhou China

**Keywords:** CD14^+^FOS^+^ monocyte, CD14^+^S100A12^+^ monocyte, inflammation, myasthenia gravis, scRNA‐seq

## Abstract

**Objectives:**

As an autoimmune disorder, myasthenia gravis (MG) manifests as an autoimmune attack on postsynaptic neuromuscular junction proteins by pathogenic autoantibodies. This immune attack disrupts neurotransmission, resulting in fatigable skeletal muscle weakness with diurnal fluctuation. However, functional cure biomarkers for patients remain limited.

**Methods:**

Peripheral blood collection was performed at three time points in patients with MG: before treatment (Pre), 1 month after treatment (Post) and functional cure (long‐term follow‐up, LF). Single‐cell RNA sequencing was performed. The clinical examination results were collected and summarised.

**Results:**

In general, patients with MG exhibited dynamic changes in immune cell composition and inflammatory features. In particular, monocytes were enriched in the LF group, and further subgroup analysis revealed enrichment of CD14^+^S100A12^+^ monocytes and depletion of CD14^+^FOS^+^ monocytes in the LF group. Moreover, inflammation scores were significantly different in the Pre, Post and LF groups.

**Conclusion:**

Our study provides a comprehensive cell landscape for patients with MG, identifies two dysregulated monocytes, elucidates the inflammation status and offers a new perspective on understanding the aetiology of functional cure and potential therapeutic strategies for patients with MG.

## Graphical abstract

Our study provides a comprehensive cell landscape for patients with myasthenia gravis (MG), discovers two dysregulated monocytes, elucidates inflammation status and offers a new perspective on understanding the aetiology of functional cure as well as potential therapeutic strategies for patients with MG.

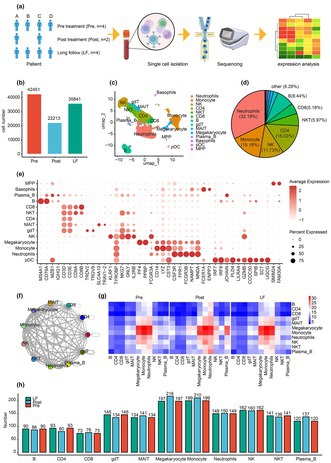

## Introduction

As a chronic neuromuscular junction autoimmune disorder,^1^ myasthenia gravis (MG) is featured by fatigable muscle weakness, with the best function occurring during the morning hours and the worst in the evening. With the typical symptoms of intermittent ptosis and diplopia, patients exhibit a wider‐ranging weakness and fatigue manifestations.^2^ MG results from pathogenic autoantibodies targeting three key postsynaptic neuromuscular junction proteins: the muscle‐type nicotinic acetylcholine receptor (AChR), muscle‐specific kinase (MuSK) protein or lipoprotein receptor‐related protein 4 (LRP4).^2^ Ocular and anti‐LRP4 MG typically present with milder clinical manifestations, whereas MuSK MG tends to exhibit a more severe phenotype.^3^ A variety of therapies have been expanded for MG clinical treatment; a significant subset of patients (approximately 33%), however, responds inadequately to treatment and suffers from recurrent exacerbations.^4^ Because of the refractoriness and recurrence of MG, understanding biomarkers in functional cure patients is particularly important.

Single‐cell RNA sequencing (scRNA‐seq) has been applied to characterise unique cell subsets in MG, including rituximab‐resistant B cells,^5^ pathogenic antibody‐producing B cells,^6^ dysregulated T helper (Th) cells^7^ and VISTA^+^ monocytes.^8^ These results confirm the diverse cell types involved in MG; however, few studies have focussed on the status of these cells in patients who achieve functional cure. Jiang et al. showed a rituximab‐resistant B‐cell spectrum in patients with MG relapses^5^; Tian et al. found that CAR T‐cell immunotherapy resulted in B‐cell lineage reconstitution and improved therapeutic efficacy.^9^ A case report in MG revealed that granulocyte macrophage colony‐stimulating factor treatment could ameliorate disease by effecting Treg cells, but the therapeutics are still under development.^10^ Consequently, it is essential to reveal the immune cell status and dysregulated monocytes in MG patients who achieve functional cure.

In our study, we performed scRNA‐seq and first analysed the cell immune status and dysregulated monocytes in patients with MG before treatment (Pre), 1 month after Methylprednisolone and Pyridostigmine Bromide treatment (Post), and functional cure (long‐term follow‐up, LF). We investigated markers over a long period of follow‐up, providing a new perspective on MG.

## Results

### Integrated analysis of MG scRNA‐seq data

**Figure 1:**
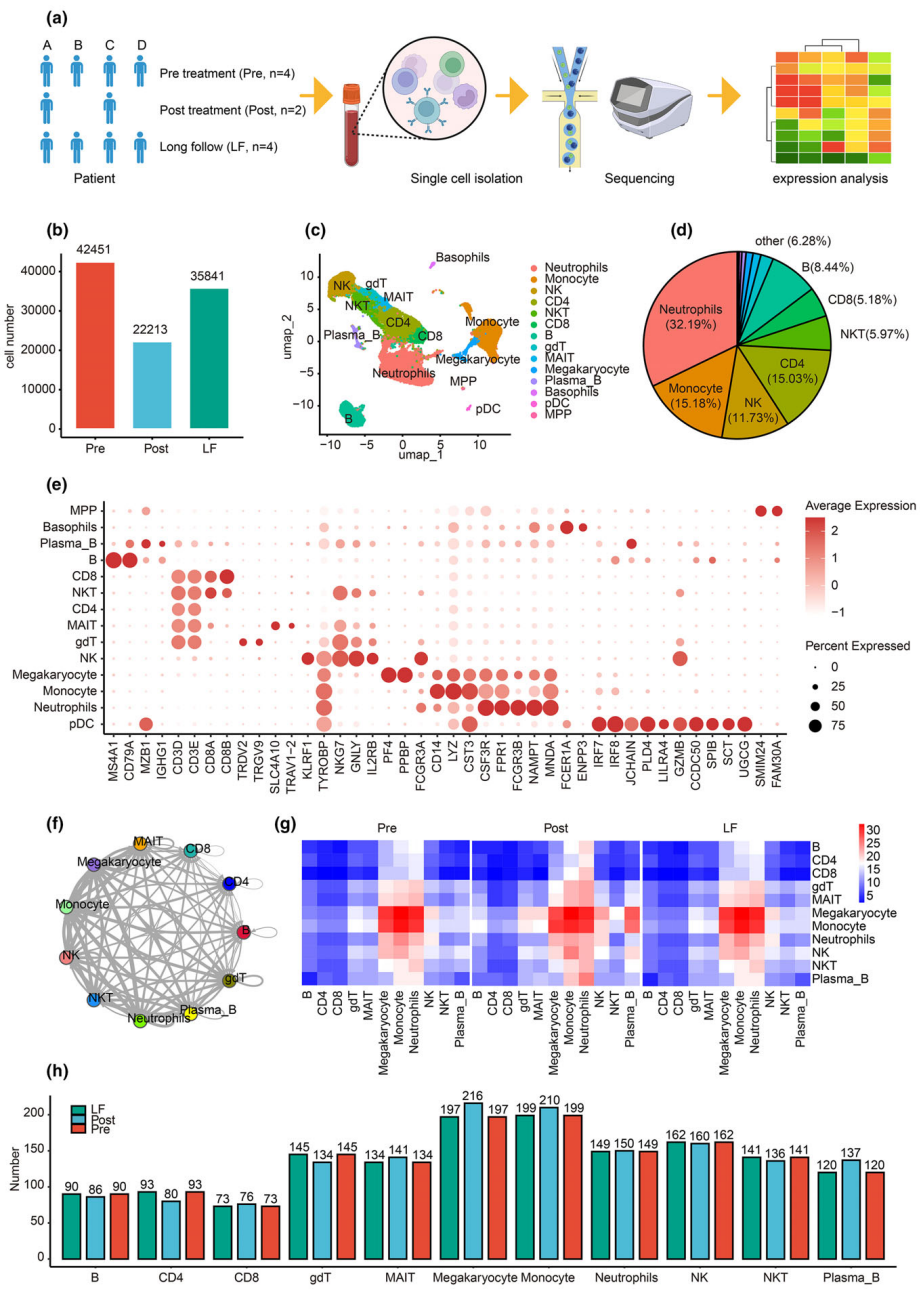
Study schematic diagram and overall results for patients with MG. **(a)** Transcriptome profiling and analysis of scRNA data of individuals with MG were performed at different time points. **(b)** Bar plot of qualified cell numbers for each group. The groups are labelled using different colours. **(c)** The 14 major cell populations from 10 samples. Each point means a cell; each colour means a cell subtype. **(d)** The pie plots for cell type percentage statistics, cell type, and percentage are labelled. **(e)** The dot plot of canonical marker genes and signature genes across cell types. **(f)** Significant ligand–receptor interactions count across these 10 major cell types. Line width represents significant ligand–receptor interaction pair count, and the arrow denotes the direction of interaction from one cell type to another. Each cell type is labelled with a single colour. A *one‐sided permutation test* defined statistical significance at *P*‐value < 0.05. **(g)** Heatmap for significant interactions numbers between ligand‐receptor: among the 10 major cell types in the Pre, Post and LF groups. A *one‐sided permutation test* defined statistical significance at *P*‐value < 0.05. Red indicates higher significant interaction number, green indicates lower significant interaction number. **(h)** Bar plot of significant interactions of ligand–receptor pair numbers among major cell types in the Pre, Post, and LF groups. Each group is labelled with a single colour. A *one‐sided permutation test* defined statistical significance at *P*‐value < 0.05. gdT, gamma‐delta T cells; LF, long‐term follow‐up when myasthenia gravis was stable or disappeared (more than half a year or 1 year); MAIT, mucosal‐associated invariant T cells; MG, myasthenia gravis; MPP, multipotent progenitors; NK, natural killer cells; NKT, natural killer T cells; pDC, monocyte‐dendritic cell progenitor cells; Post, 1 month after treatment; Pre, before treatment.

ScRNA transcriptome profiles were obtained from patients with MG at three time points: before treatment (Pre), 1 month after treatment (Post) and at long‐term follow‐up when MG was stable or disappeared (more than half a year or 1 year; LF) (Figure 1a). The clinical features and laboratory results of the patients with MG were summarised (Table 1). The counts of Unique Molecular Identifier (UMI), gene numbers, mitochondrial genes, ribosomal genes, immunoglobulin genes, HLA genes and erythrocyte‐related genes were calculated to identify abnormal expression patterns in each sample (Supplementary figure 1a). The percentage of ribosomal genes was relatively high in samples of the Pre, Post and LF groups, indicating that all ribosomal genes were excluded for variable gene identification. After filtering low‐quality cells (criteria in the Methods section), we obtained 100 505 cells from 10 samples, with a range of 7179 to 13 195 cells per sample (Supplementary figure 1b), including 42 451 cells in the Pre group, 22 213 in the Post group and 35 841 in the LF group (Figure 1b).

**Table 1:** Clinical features of enrolled patients

Patient	Ophthalmic phenotype	Neostigmine test	Antibody	Mediastinal imaging	Treatment	Time point of symptom improvement	Follow up
1	Right ptosis, convergence insufficiency	Positive	MuSK‐Ab	No abnormalities	Methylprednisolone, pyridostigmine bromide	2 weeks	Methylprednisolone discontinued 6 months later. No eye or systemic symptoms when 12 months after treatment
2	Bilateral ptosis, diplopia, convergence insufficiency	Positive	AchR‐Ab	Mediastinal thymic tissue	Methylprednisolone, pyridostigmine bromide	3 weeks	Methylprednisolone 5 mg qd, Pyridostigmine Bromide 20 mg tid 12 months. No abnormal symptoms when 12 months follow up
3	Right ptosis	Positive	AchR‐Ab	Mediastinal thymic tissue	Methylprednisolone, pyridostigmine bromide	1 week	Pyridostigmine Bromide 30 mg tid for 12 month. No eye or systemic symptoms observed
4	Left ptosis, left lateral gaze restriction	Positive	AchR‐Ab	No abnormalities	Methylprednisolone, pyridostigmine bromide	1 week	5 months stop al medicine. No abnormal symptoms when 12 months follow up

Fourteen major cell types (Figure 1c), including neutrophils, monocytes, CD8^+^ T cells, CD4^+^ T cells, gamma‐delta T cells (gdT), mucosal‐associated invariant T cells (MAIT), B cells, plasma B cells, megakaryocytes, natural killer (NK) cells, natural killer T (NKT) cells, basophils, plasmacytoid dendritic cell (pDC), and multipotent progenitors (MPP), were annotated based on canonical markers and collected genes. Neutrophils accounted for more than 32.19% of all the cells, followed by monocytes (15.18%), CD4^+^ T cells (15.03%), NK cells (11.73%) and B cells (8.44%) (Figure 1d). All cell types expressed specific canonical marker genes (Figure 1e).

To investigate cellular crosstalk, ligand–receptor interactions among major immune subpopulations were systematically evaluated and all significant interactions were obtained (Figure 1f). A relatively high connection between monocytes, megakaryocytes, neutrophils and other cells was observed (Supplementary figure 1c, d). However, the overall significant number of interactions among the Pre, Post and LF groups showed similar patterns (Figure 1g and h).

### Association of disease status with dynamic changes of immune cell compositions

**Figure 2:**
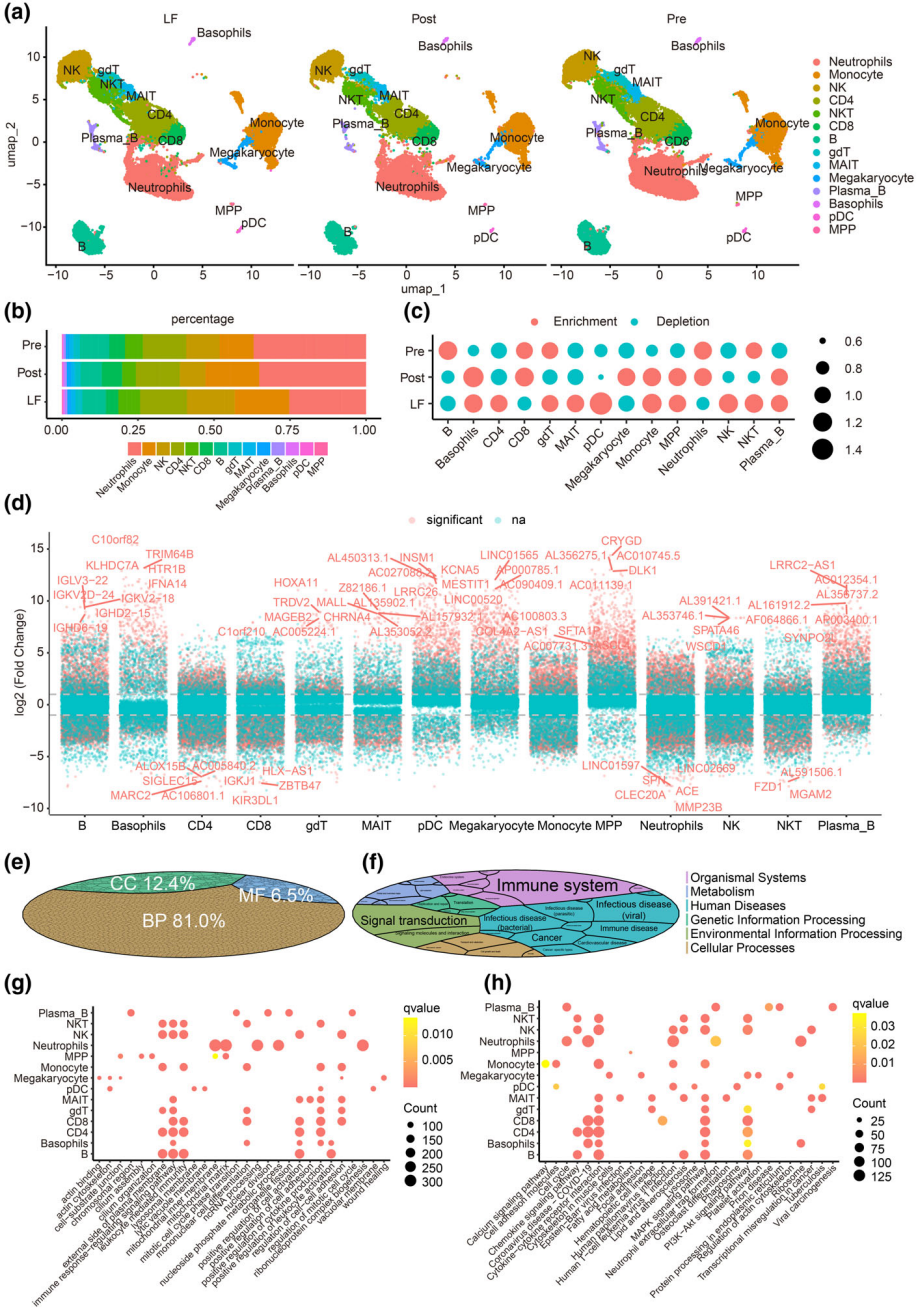
Association of disease status with dynamic changes of immune cell compositions. **(a)** Fourteen major cell types in the Pre, Post, and LF groups. Each point represents a cell, and each colour represents a specific cell type. **(b)** Bar plot of the percentage of cell types among the Pre, Post and LF groups. Each colour represents a specific cell type. **(c)** Dot plot of the ratio of observed/expected numbers of cell types among the Pre, Post, and LF groups. A ratio > 1 is labelled red, indicating enrichment; a ratio < 1 is labelled green, indicating depletion. **(d)** Significantly altered genes for each cell type. The top five genes with the highest fold change were labelled for each type. Red indicates significantly altered genes, green indicates non‐significantly altered genes, and each point represents one gene. **(e)** The GO terms classification for all significantly altered genes. **(f)** The percentage of pathways classification in the KEGG database for all significantly altered genes. **(g)** Enrichment analysis of GO terms for each cell type in patients with MG. **(h)** Enrichment analysis of KEGG pathways for each cell type in patients with MG. gdT, gamma‐delta T cells; LF, Long‐term follow‐up when myasthenia gravis was stable or disappeared (more than half a year or 1 year); MAIT, mucosal‐associated invariant T cells; MG, myasthenia gravis; MPP, multipotent progenitors; NK, natural killer cells; NKT, natural killer T cells; pDC, monocyte dendritic cell progenitor cells; Post, 1 month after treatment; Pre, before treatment.

To investigate the distribution of major cells according to MG status, we split all cells into the Pre, Post and LF groups and observed a highly similar distribution for all major cell types (Figure 2a). The relative percentages of each major cell type within individual samples were calculated (Figure 2b). In the LF group, the relative percentage of neutrophils decreased, whereas that of monocytes, NK cells, and CD4 T cells increased (Figure 2b). The ratio of observation to expectation also confirmed the depletion of neutrophils/CD8 T cells and the enrichment of monocyte/CD4 T cells/NK cells in the LF group (Figure 2c). Notably, immune cells (including CD4^+^T cells, CD8^+^T cells, NK cells, MAIT cells, B cells and neutrophils) dynamically changed in the LF group, indicating a good immune status for patients with MG. These data revealed the dynamic changes of immune cell profiles among individuals in Pre, Post and LF groups.

To explore the features of genes and pathways in MG‐related cell types, we compared gene expression differences among these cells and identified significantly altered genes. Those with the highest fold change and significant *P‐*value (adjusted *P‐*value < 0.05) were labelled (Figure 2d). All significantly altered genes involved in Gene Ontology (GO) terms and pathways were analysed. Statistical analysis of all significant GO terms revealed 12.4% of terms in cellular component, 81.0% of terms in biological process and 6.5% of terms in molecular function (Figure 2e). Kyoto Encyclopedia of Genes and Genomes (KEGG) pathway assay revealed that these genes involved in immune, signal transduction and infectious disease pathways (Figure 2f), indicating an association between these MG‐featured genes and the immune status in patients with MG. The top five terms for each cell type were extracted for visualisation, with the majority enriched in immune functions, including immune response‐regulating signalling, leukocyte‐mediated immunity, cell activation, cytokine production and leukocyte activation (Figure 2g). The top five pathways for each cell type also highlighted the immune and metabolic pathways, including chemokine signalling, cytokine–cytokine receptor interaction, MAPK signalling and PI3K‐Akt signalling pathways (Figure 2h).

### Expression profiles of monocytes in patients with MG

**Figure 3:**
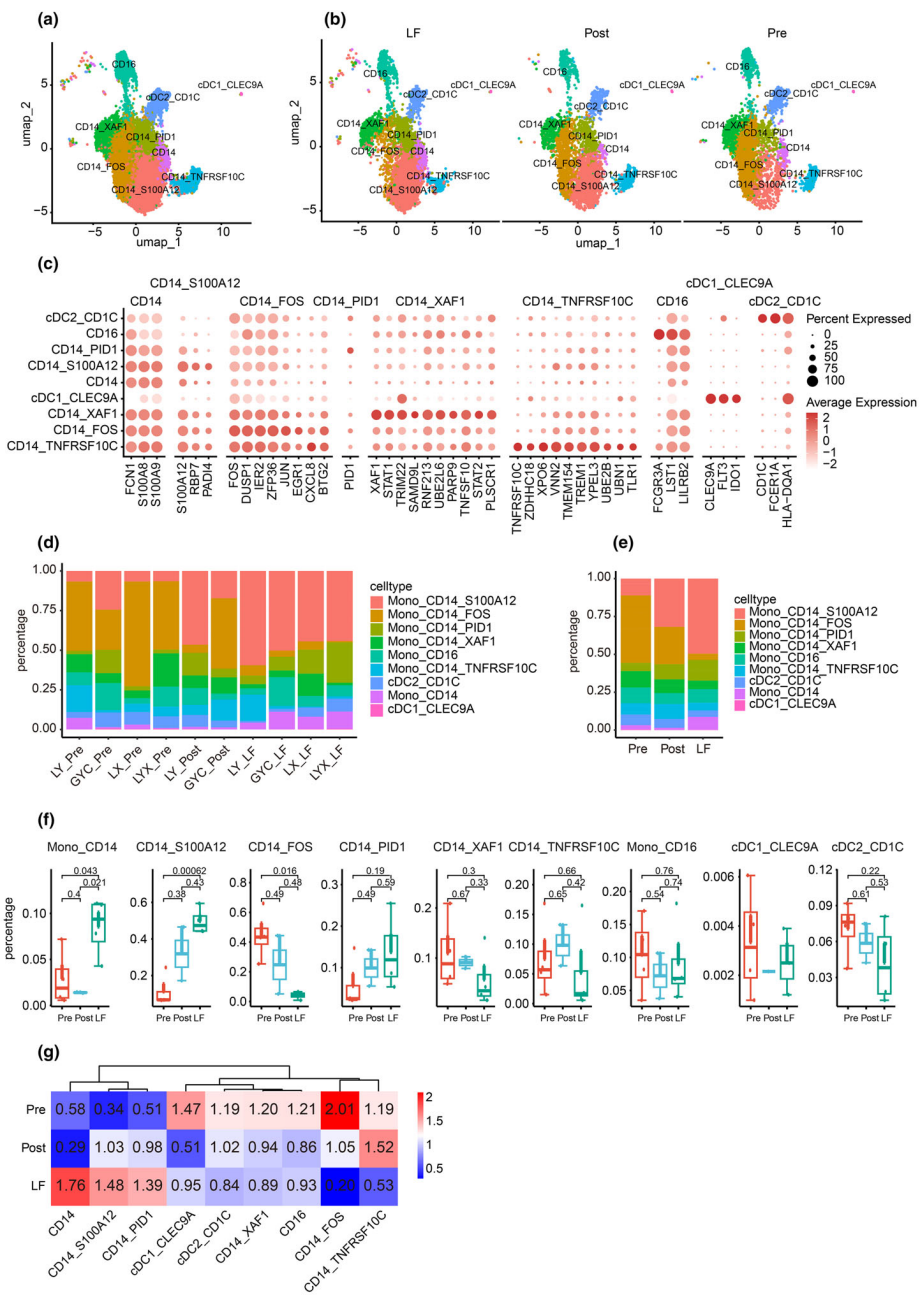
Expression profiles of monocytes in patients with MG. **(a)** Nine major monocyte subtypes. Each point represents a cell, and each colour represents a subtype. **(b)** Nine major subtypes of monocytes among the Pre, Post, and LF groups. Each point represents a cell, and each colour represents a specific cell type. **(c)** Dot plot of canonical marker and signature genes across cell types. **(d)** The pie plots for cell type percentage statistics in the samples, cell type, and percentage are labelled. **(e)** The pie plot for cell type percentage statistics among the Pre, Post and LF groups, cell type, and percentage are labelled. **(f)** Bar plot for cell type percentage statistics among the Pre, Post and LF groups. Data are analysed by *Wilcoxon‐test*. The *P*‐values for each comparison are labelled. **(g)** Heatmap of the ratio of observed/expected numbers of cell types among the Pre, Post, and LF groups. Enrichment trends are clustered among cell types. A ratio > 1 is labelled red, indicating enrichment; a ratio < 1 is labelled blue, indicating depletion. gdT, gamma‐delta T cells; LF, long‐term follow‐up when myasthenia gravis was stable or disappeared (more than half a year or 1 year); MAIT, mucosal‐associated invariant T cells; MPP, multipotent progenitors; NK, natural killer cells; NKT, natural killer T cells; pDC, monocyte‐dendritic cell progenitor cells; Post, 1 month after treatment; Pre, before treatment. “LY”, “GYC”, “LX”, “LYX” are patients IDs.

Given the enrichment in the LF group (Figure 2c), high interaction with other cells (Figure 1g), and immune features of cell types (Figure 2e–h), monocytes were selected for further investigation of potential roles in association with long‐term follow‐up for patients with MG. Nine monocyte subtypes were annotated using classical markers and signature genes (Figure 3a). Obvious changes were observed among the Pre, Post and LF groups, especially in CD14^+^FOS^+^ monocytes and CD14^+^S100A12^+^ monocytes (Figure 3b). Generally, all monocytes were grouped into two classical dendritic cells (cDCs), including cDC1_CLEC9A (highly expressed CLEC9A, FLT3, and IDO1) and cDC2_CD1C (highly expressed CD1C, FCGR1A, and HLA‐DQA1), CD16^+^ monocytes (highly expressed FCGR3A, LST1, and LILRB2) and CD14^+^ monocytes (highly expressed FCN1, S100A8 and S100A9) in all three groups. CD14^+^ monocytes were further grouped into five subtypes based on the feature genes: CD14^+^S100A12^+^ monocytes highly expressed S100A12, RBP7 and PADI4; CD14^+^FOS^+^ monocytes shared markers of CD14^+^S100A12^+^ and highly expressed FOS, DUSP1, IER2, ZFP36, JUN, EGR1 CXCL8, and BTG2; CD14^+^PID1^+^ monocytes highly expressed PID1; CD14^+^XAF1^+^ monocytes shared markers of CD14^+^S100A12^+^ and CD14^+^FOS^+^ monocytes and highly expressed XAF1, STAT1, TRIM22, SMAD9L, RNF213, UBE2L6, PARP9, TNFSF10, STAT2 and PLSCR1; and CD14^+^TNFRSF10C^+^ monocytes shared markers of CD14^+^S100A12^+^ and CD14^+^FOS^+^ monocytes and highly expressed TNFRSF10C, ZDHHC18, XPO6, VNN2, TMEM154, TREM1, YPEL3, UBE2B, UBN1 and TRL1 (Figure 3c). The relative percentages of cell types revealed an obvious expansion of CD14^+^S100A12^+^ monocytes and depletion of CD14^+^FOS^+^ monocytes in the LF group (Figure 3d and e). Statistical comparison showed a significant expansion of CD14^+^ and CD14^+^S100A12^+^ monocytes and a significant depletion of CD14^+^FOS^+^ monocytes in the LF group (Figure 3f). The ratio of observation to expectation also confirmed the enrichment of CD14^+^ and CD14^+^S100A12^+^ monocytes and depletion of CD14^+^FOS^+^ monocytes in the LF group (Figure 3g).

**Figure 4:**
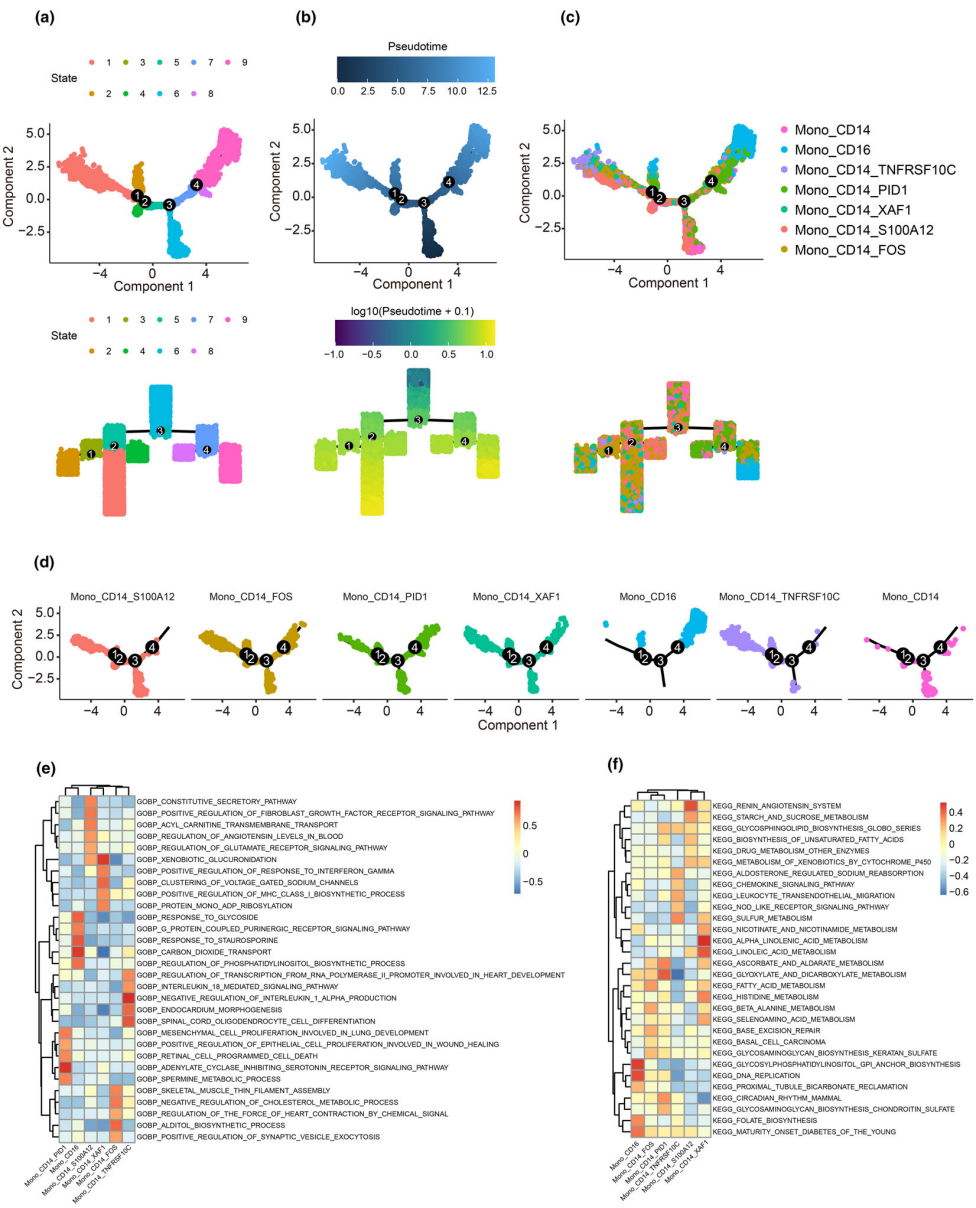
Pseudotime trajectory and pathways for monocytes. **(a)** Pseudotime‐ordered analysis of monocytes in MG samples. Up: The scatter plot of pseudotime is labelled from the beginning (dark blue) to the end (light blue), with each point representing one cell. Down: The tree plot of pseudotime is labelled from the beginning (dark blue) to the end (light yellow), with each point representing one cell. **(b)** Pseudotime status analysis of monocytes from MG samples. Up: Scatter plot of status for all cells, where each point represents one cell. Down: Tree plot of status, where each point represents one cell. **(c)** Pseudotime analysis of monocytes from MG samples labelled by cell type. Up: Scatter plot of cell type for all cells, where each point represents one cell. Down: Tree plot of cell type, where each point represents one cell. **(d)** Pseudotime analysis of monocytes from MG samples split by cell type. **(e)** Enrichment analysis of GO terms for each monocyte cell type in patients with MG. Red indicates higher values of activation, while blue indicates inhibition. **(f)** Enrichment analysis of KEGG pathways for each monocyte cell type in patients with MG. Red indicates higher values of activation, while blue indicates inhibition.

To explore the cell transitions among these monocytes, we constructed a pseudotime trajectory, and trees showed four major branches (Figure 4a). Pseudotime trajectory analysis is used for identifying cell transition. Briefly, CD14^+^TNFRSF10C^+^ monocytes andCD16^+^ monocytes were located at the terminal point of cell evolution on this map, suggesting a three‐branched structure. Other cells located at the branch point of the pseudotime trajectory exhibit a transitional state, indicating these cells possess fate plasticity (Figure 4b–d). The top five enrichment GO terms and KEGG pathways among monocyte subtypes were determined using gene set variation analysis. Specific GO term enrichment patterns were observed among subtypes, especially alpha‐adrenergic receptor binding, interferon receptor binding and L‐proline transmembrane transporter activity terms in CD14^+^FOS^+^ monocytes, and lactate dehydrogenase activity, oestrogen alpha hydroxylase activity and haemoglobin binding terms in CD14^+^S100A12^+^ monocytes (Figure 4e). The top five KEGG pathways revealed metabolic status: glycosphingolipid biosynthesis ganglio, glycan degradation, renin‐angiotensin system, and starch and sucrose metabolism pathways in CD14^+^S100A12^+^ monocytes, and glycosaminoglycan degradation, glycosaminoglycan biosynthesis, heparan sulfate and regulation of autophagy pathways in CD14^+^FOS^+^ monocytes (Figure 4f).

### Inflammation pathways among cell types and time points

**Figure 5:**
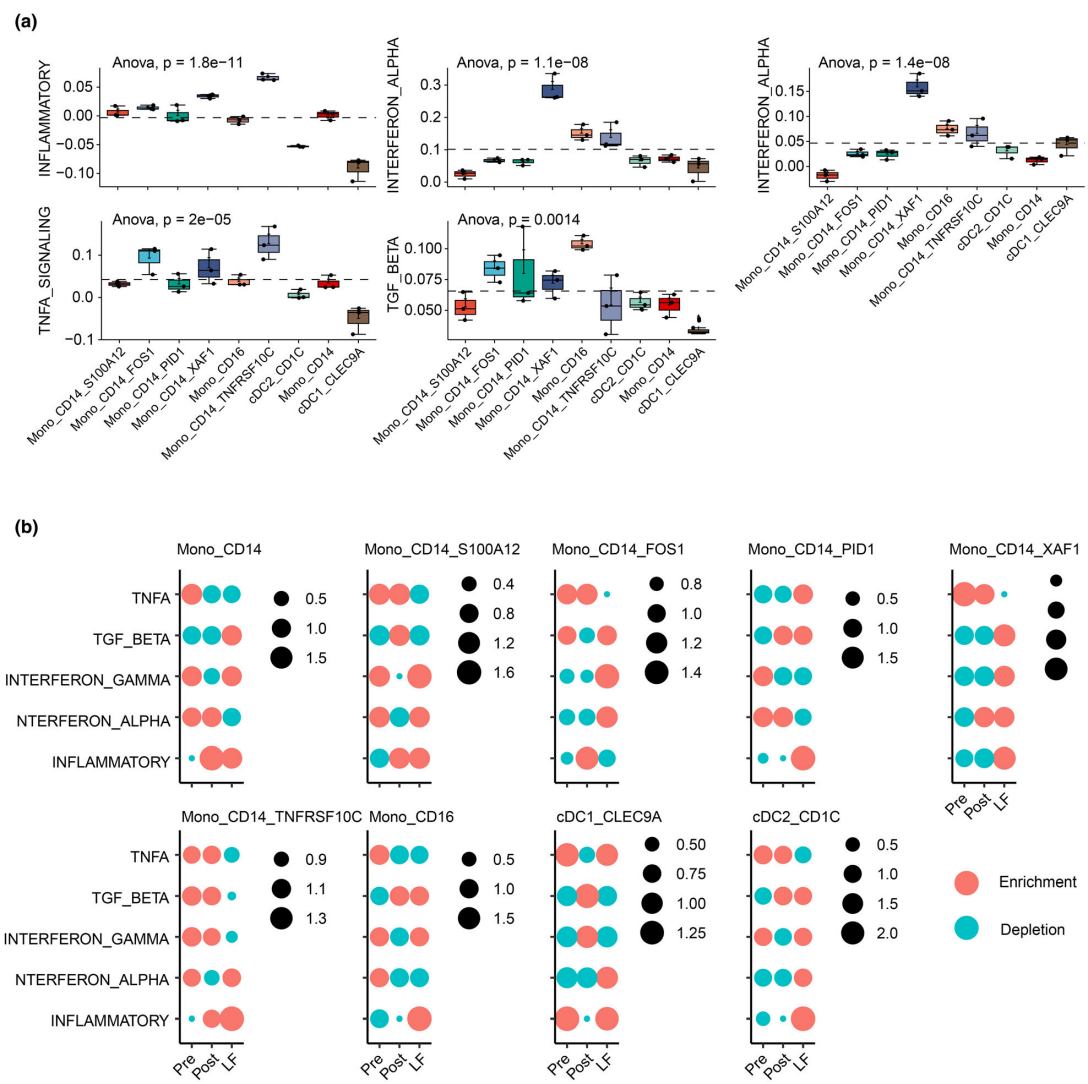
Inflammation pathways among cell types and time points. **(a)** Box plot for the average scores of inflammation pathways. Data are analysed by *ANOVA*. The overall *P*‐value is labelled. **(b)** Dot plot of the ratio of observed/expected score of pathways among the Pre, Post and LF groups. A ratio > 1 is labelled red, indicating enrichment; a ratio < 1 is labelled green, indicating depletion. Pre, before treatment; Post, 1 month after treatment; LF, long‐term follow‐up when myasthenia gravis was stable or disappeared (more than half a year or 1 year).

Considering the inflammatory immune status in patients with MG (Figures 2e–h and 3d and e), we investigated the inflammation pathways in MSigDB (details in the Methods section). Generally, the inflammatory score of CD14^+^TNFRSF10C^+^ and CD14^+^XAF^+^ monocytes, the interferon alpha score of CD14^+^XAF^+^ monocytes, the interferon gamma score of CD14^+^XAF^+^ monocytes, and the TNF alpha score of CD14^+^FOS^+^ and CD14^+^TNFRSF10C^+^ monocytes were significantly higher than those of other monocytes (Figure 5a). To investigate these five pathways for each monocyte subtype among the Pre, Post and LF groups, we further compared them at different time points (Figure 5b). Particularly, the LF group had significantly higher scores of CD14^+^S100A12^+^ monocytes in all five pathways than the Pre group (Figure 5b, left panel). In addition, CD14^+^FOS^+^ monocytes showed a significantly higher score of interferon alpha/gamma (IFN‐α/γ) and a lower score of TNF alpha (Figure 5b, second left panel). CD14^+^PID1^+^ monocytes exhibited a significantly higher score in inflammatory, TGF‐beta and TNF‐alpha (TNF‐α) pathways (Figure 5b, third left panel). CD14^+^XAF1^+^ monocytes exhibited a significantly lower score in IFN‐α/γ and TNF‐α pathways (Figure 5b, fourth left panel). Finally, CD14^+^TNFRSF10C^+^ monocytes exhibited a significantly lower score in all pathways except for inflammatory response.

## Discussion

Myasthenia gravis is a chronic autoimmune disorder of the neuromuscular junction.^1^ Muscle weakness is the predominant manifestation, typically worsening with repeated muscle activity. Muscle function is usually optimal during the morning hours, with more worsening status at the end of the day. The extraocular muscles are commonly affected asymmetrically, with typical symptoms of intermittent ptosis and diplopia. Generalised MG involves more widespread weakness and fatigability, including bulbar, limb and neck muscles.^2^

Autoantibodies targeting functionally key molecules located on the postsynaptic membrane of neuromuscular junctions including AChR, MuSK and LRP4 drive the autoimmune pathogenesis of MG. Most MG patients (80–85%) possess anti‐AChR IgG, while a minority (4–8%) are seropositive for anti‐MuSK IgG.^2^ Additionally, RP4‐specific antibodies have been detected in patient subsets, with subgroups classified based on clinical phenotypes, age at onset, presence of autoantibody patterns and thymus pathological features.^1^ These subgroups exhibit distinct characteristics in epidemiological profiles, pathogenic mechanisms, clinical severity and therapeutic response, facilitating the guidance of personalised treatment strategies.^11^ Notably, ocular MG and anti‐LRP4 antibody‐positive MG typically demonstrate milder manifestations, whereas MuSK‐positive MG and presumably thymoma‐associated MG often present with more severe phenotypes.^3^

ScRNA‐seq technology enables us to investigate RNA expression in single‐cell markers based on highly expressed markers, which are annotated into specific populations, such as immune cells and epithelial cells. By analysing dynamic changes in cell populations, functional cells were discovered and defined, such as the exhausted state of T cells,^12^ inflammatory monocytes^13^ and antibody‐secreting B cells.^5^ For MG, studies focussed on specific cell subtypes, including rituximab‐resistant B cells during MG relapses,^5^ producing nAChR‐Abs B cells,^6^ upregulation of anti‐AChR antibodies associated with CD180‐ B cells,^14^ dysregulated inflammatory circulating memory Th cells^7^ and dysregulation of VISTA^+^ monocytes.^8^

Monocytes are key innate immune cells which are involved in developmental MG. A study by Fan et al. identified the VISTA^+^ monocytes in MG.^8^ A Mendelian randomisation study identified that upregulation of CD40, HLA‐DR and CD64 of monocyte cells is promoting MG development.^15^ Notably, consistent with previous studies, our study further validated that dysregulation of immune cells, especially monocytes, is likely to contribute to MG development. In contrast, this study further focussed on the dynamic changes of monocyte subtypes in the Pre, Post and LF groups. We observed the dynamic changes of monocyte subtypes and found that monocytes were enriched in the LF group (Figure 2c), with high interactions with other cells (Figure 1g) and immune features (Figure 2e–h). These findings demonstrate dysregulated monocytes in patients with MG.

S100A12, a member of the S100 family of EF‐hand calcium‐binding proteins, is involved in multiple cellular processes^16^ and is linked to certain autoimmune reactions as a part of the innate immune response.^17^ S100A12 is considered a crucial factor in inflammation and is related to inflammatory processes in autoimmune disorders. The protein level of S100A12 was significantly increased during ongoing inflammation in rheumatoid arthritis.^18^ Several studies have demonstrated that the levels of S100A12 are increased in patients with MG.^19^, ^20^ The expression of S100A12 is enhanced by TNF‐α and IL‐6 in MG.^20^ Our analysis revealed enrichment of CD14^+^S100A12^+^ monocytes in the LF group based on the classic and feature markers (Figure 3d and e), indicating altered immune‐inflammatory status throughout treatment. The functional mechanism of S100A12 in MG requires further investigation.

FOS was a part of transcription factor (TF) complex for activator protein 1 (AP‐1).^21^ Fra1, also a FOS part of AP‐1 TF, showed significantly increased expression in MG thymus.^22^ Recently, low expression of FOS was observed after Jianpi Yiqi Bugan Yishen Decoction treatment in patients with MG.^23^ Consistent with their results, we observed the depletion of CD14^+^FOS^+^ monocytes in the LF group, demonstrating the key role of FOS in MG therapy.

This study comprehensively compares the dynamic evolution of the immune cell landscape in MG patients throughout the treatment timeline and identifies two dysregulated monocytes. Furthermore, we elucidate the progressive inflammation status. These findings provide new evidence for the development of therapeutic strategies and propose potential biomarkers for assessing treatment efficacy and prognosis for patients with MG.

Several limitations need to be acknowledged. Our study primarily relies on transcriptomic data. While this approach provides high‐resolution characterisation of cellular states, it lacks direct validation of key findings at the protein level. Further studies should quantify proportional changes in these monocyte subsets through protein‐level detection of key markers. This is essential to more reliably assess their biological functions and clinical significance.

### Summary

In our study, we investigated single‐cell RNA sequencing data from patients with MG at three time points and found that monocytes were enriched in the LF group, especially the enrichment of CD14^+^S100A12^+^ monocytes and depletion of CD14^+^FOS^+^ monocytes. Our study identified two dysregulated monocytes, elucidated inflammation status, and provided insights on understanding the aetiology of functional cure in patients with MG.

## Methods

### Sample collection

#### Ethics statement

This study was approved by the ethics committee of the Second Affiliated Hospital of Zhejiang University (No. 2024.0973). All participants provided written informed consent prior to sample collection, and all procedures strictly followed the principles of the Declaration of Helsinki.

#### Collection of peripheral blood mononuclear cell samples

Four patients newly diagnosed with MG at the Second Affiliated Hospital of Zhejiang University were included. Peripheral blood samples were collected at three stages: before treatment, 1 month after treatment, and at long‐term follow‐up when MG was stable or had disappeared (more than half a year or 1 year). The clinical and demographic features were summarised (Table 1). All samples were subjected to single‐cell transcriptome sequencing utilising a Singleron platform.

### scRNA‐seq and raw data generation

The PBMCs were collected as per the previous description.^24^ In brief, the blood sample was gently stratified onto Ficoll‐Paque Plus medium (GE Healthcare, Uppsala, Sweden) in a centrifuge tube and centrifuged by density gradient centrifugation. The red blood cells were removed by lysis buffer according to the instructions (Singleron, Nanjing, China). The solution was centrifuged and suspended in PBS (HyClone, Utah, USA). Subsequently, peripheral blood samples underwent centrifugation, and the supernatant was discarded. Following the removal of red blood cells and isolation by centrifugation, PBMCs were washed thoroughly with PBS, producing a single‐cell suspension suitable for scRNA‐seq.

Cell suspensions (2 × 10^5^ mL^−1^) were processed through Singleron Matrix® Single Cell Processing System (Singleron, Beijing, China). Following barcoded bead capture, the captured mRNA underwent reverse transcription, cDNA synthesis and PCR amplification. Following fragmentation, amplified cDNA underwent ligation with sequencing adapters. The sequencing libraries were obtained,^25^ and then sequenced on the NovaSeq 6000 platform (Illumina, USA).

### scRNA‐seq data analysis

CeleScope (v1.5.2, https://github.com/singleron‐RD/CeleScope) was used for generating the expression matrix from raw sequencing reads with default parameters.

RNA expression matrix quality control, cell filtering, clustering and analysis were performed using Seurat (v.5.0.0)^26^ following the official guidelines. The percentages of mitochondrial, ribosomal, immunoglobulin, human leukocyte antigen (HLA) and erythrocyte‐related genes were calculated for all samples. Cells underwent quality control using three step‐by‐step metrics: UMI counts, gene numbers and mitochondrial gene proportion. Specifically, a combination of UMIs (< 25,000), gene counts (500 < genes < 5000), and mitochondrial genes (percentage < 20) were utilised for raw gene‐cell‐barcode matrix filtering. After low‐quality cells were removed from the matrix, the expression for each cell was normalised via ‘LogNormalize’. The data were multiplied by a scale factor (10 000) and log‐transformed the result. The FindVariableFeatures function was used to identify the genes with the most obvious cell‐to‐cell differences, and a consensus list of 2000 variable genes was obtained. Linear transformation was performed using variable genes (excluding all ribosomal and mitochondrial genes) with the greatest recovery rates across samples. The principal components (PCs) were computed using RunPCA, with significant PCs selected via JackStraw and PCElbowPlot functions of Seurat.

Batch‐effect correction among the samples was performed using a harmony algorithm.^27^ The PCA matrix was generated with 50 PCs using selected genes and then analysed by FindClusters function (resolution: 0.8). A total of 31 clusters were obtained. Subsequently, 17 cell populations including B cells (‘MS4A1’, ‘CD79B’), plasma B cells (‘MZB1’, ‘IGHG1’), CD4^+^ T cells (‘CD3D’, ‘CD3E’), CD8^+^ T cells (‘CD8A’, ‘CD8B’), NK cells (‘KLRF1’, ‘NKG7’, ‘GNLY’), NKT cells (‘CD3D’, ‘CD3E’, ‘CD8A’, ‘CD8B’, ‘NKG7’, ‘GNLY’), MAIT cells (‘CD3D’, ‘CD3E’, ‘SLC4A10’, ‘TRAV1‐2’), gdT cells (‘CD3D’, ‘CD3E’, ‘TRDV2’, ‘TRGV9’), megakaryocytes (‘PF4’, ‘PPBP’), monocytes (‘CD14’, ‘LYZ’, ‘CST3’), neutrophil cells (‘CSF3R’, ‘FPR1’, ‘FCGR3B’, ‘NAMPT’, ‘MNDA’), PDC cells (‘IRF7’, ‘IRF8’, ‘JCHAIN’, ‘PLD4’, ‘GZMB’, ‘CCDC50’, ‘SPIB’, ‘SCT’, ‘UGCG’), MPP (‘SMIM24’, ‘FAM30A’) and basophils (‘FCER1A’, ‘ENPP3’) were annotated by classic and variable genes. The progenitor cell markers were obtained from a previous study.^28^ To identify clusters within the cell types of interest, a second round of monocyte clustering was performed. Like the first round, starting from raw counts, as described above, with a resolution of 0.5. Subclusters retained ≥ 30 significantly upregulated genes (FDR < 0.05, log_2_FC > 1) versus other cells.

### Pathway analysis

All significantly changed genes (FDR < 0.05, |log_2_FC| > 1) were obtained among the cell types. The ClusterProfiler (v.4.1.10, https://github.com/YuLab‐SMU/clusterProfiler) was used to analyse the enrichment of GO terms and KEGG pathways in these cells, and significantly altered pathways (*q*‐value < 0.05) were obtained. The top five most significant pathways (with the lowest *q* values) were selected to determine the expression patterns of each cell type.

### Inflammation‐related scores of specific cell populations

Defined genes extracted from MSigDB were utilised for calculating the overall score of each cell type.^29^ The inflammation‐related scores were defined using the gene list from the following terms in MSigDB: inflammation response score, ‘HALLMARK_INFLAMMATORY_RESPONSE’; interferon alpha response, ‘HALLMARK_INTERFERON_ALPHA_RESPONSE’; interferon gamma response, ‘HALLMARK_INTERFERON_GAMMA_RESPONSE’; TGF beta signalling score, ‘HALLMARK_TGF_BETA_SIGNALING’; and TNF alpha score, ‘HALLMARK_TNFA_SIGNALING_VIA_NFKB’. Cell states were scored based on the mean level of gene expression to the reference genes.^30^ The general distribution of inflammation scores among cell types was analysed using *analysis of variance (ANOVA)*, and comparisons among groups were statistically tested using the nonparametric *Wilcoxon‐test*.

### Trajectory analysis

Single‐cell pseudotime trajectories were obtained using the Monocle2 package.^31^ NewCellDataSet (), estimateSizeFactors() and estimateDispersions() were used for the analyses. The detectGenes() function was used to filter low‐quality cells with ‘mean_expression ≥ 0.1’.

### STARTRAC distribution

The ratio of observation/expectation was used to evaluate the distribution of cells.^32^ It was calculated according to the following formula:

$$\mathrm{Ro}/e=\frac{\text{observed}}{\text{expected}}$$

### Cell communications

Cell communications molecules were analysed via CellPhoneDB^33^ (https://www.cellphonedb.org). We conducted pairwise assessments across all identified cell types. Ligand–receptor interactions were considered biologically relevant only if expressed in > 10% of cells within a given cluster. Ligand‐receptor interactions were performed using the statistical method in CellPhoneDB with default settings.

## Author contributions

**Yufan Guo:** Conceptualization; methodology; validation; writing – original draft. **Yu Gu:** Conceptualization; methodology; validation; writing – original draft. **Yuting Jin:** Data curation; software; writing – original draft. **Xintao Wu:** Data curation; software; writing – original draft. **Yuting Lou:** Data curation; software; writing – original draft. **Pu Miao:** Data curation; software; writing – original draft. **Ye Wang:** Data curation; software; writing – original draft. **Bijun Zhang:** Data curation; software. **Xueting Lin:** Data curation; software. **Chudi Zhang:** Data curation; software. **Jianhua Feng:** Supervision; writing – original draft; writing – review and editing.

## Conflict of interest

The authors declare no conflict of interest.

## Supporting information


Supplementary figure 1

## Data Availability

The data that support the findings of this study are available from the corresponding author upon reasonable request.
