# Supplementary material for: Single‐cell RNA sequencing reveals cell immune status and dysregulated monocytes in patients with myasthenia gravis
Source: Clin Transl Immunology. 2025 Oct 5;14(10):e70052. doi: 10.1002/cti2.70052 (PMC12497684; doi:10.1002/cti2.70052)
Supplement: Supplementary file 1 — Supplementary figure 1 [file CTI2-14-e70052-s001.docx]

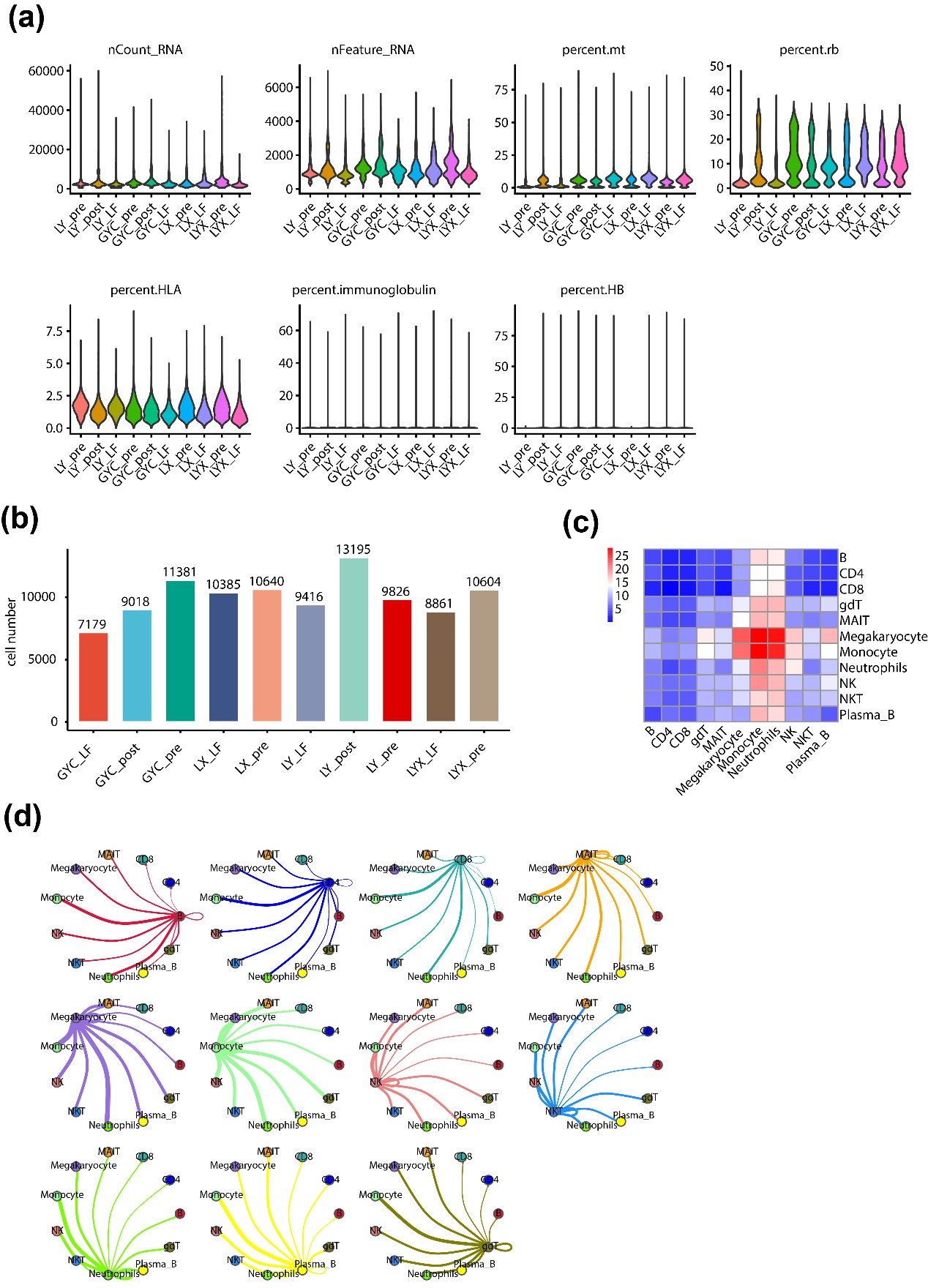


**Supplementary Figure 1** Quality control and cell-cell communication results of single‐cell sequencing for patients with MG.

**(a)** Volcano plot for the number of counts and genes, percentage of mitochondria, ribosome HLA, immunoglobulin, and erythrocyte-related genes in patients with MG.

**(b)** Bar plot of cell number for each sample in patients with MG.

**(c)** Heatmap for significant interactions numbers between ligand-receptor among the ten major cell types in all cells. A *one-sided permutation test* defined statistical significance at *P-value* < 0.05.

**(d)** Significant ligand-receptor interactions count across these ten major cell types. The line width means the significant ligand-receptor interaction numbers, and the arrow denotes the direction of interaction from one cell type to another. Each cell type is labeled with a single color. A *one-sided permutation test* defined statistical significance at *P-value* < 0.05.
